# Autoimmune and infectious encephalitis: development of a discriminative tool for early diagnosis and initiation of therapy

**DOI:** 10.1007/s00415-024-12712-7

**Published:** 2024-10-05

**Authors:** Tobias Moser, Joachim Gruber, Eirini Mylonaki, Vincent Böhm, Daniel Schwarzenhofer, Anna R. Tröscher, Eva Lenzenweger, Ingomar Krehan, Eva Söllradl, Markus Leitinger, Raimund Helbok, Eugen Trinka, Tim J. von Oertzen, Judith N. Wagner

**Affiliations:** 1 Department of Neurology, Neurocritical Care, and Neurorehabilitation, Christian Doppler University Hospital, Paracelsus Medical University and Centre for Cognitive Neuroscience, European Reference Network EpiCARE, Salzburg, Austria; 2 Department of Neurology, Kepler University Hospital, Johannes Kepler University, Linz, Austria; 3 Neuroscience Institute, Christian Doppler University Hospital, Paracelsus Medical University and Centre for Cognitive Neuroscience, Salzburg, Austria; 4 Medical Directorate, University Hospital Würzburg, Würzburg, Germany; 5 Department of Neurology, Evangelisches Klinikum Gelsenkirchen, Teaching Hospital University Duisburg-Essen, Gelsenkirchen, Germany

**Keywords:** Encephalitis, Inflammation, Infection, Differential diagnosis, Prognosis, Therapeutic management

## Abstract

**Background:**

Encephalitis originates from diverse autoimmune and infectious etiologies. Diagnostic challenges arise due to the spectrum of presentation and the frequent absence of specific biomarkers. This study aimed to comprehensively characterize and differentiate autoimmune encephalitis (AE) from infectious encephalitis (IE) in adults, and disentangle clinical, paraclinical, and therapeutic differences.

**Methods:**

A cohort study spanning 10 years was conducted across three Austrian tertiary care hospitals. Inclusion criteria comprised adults with probable or definite encephalitis. Demographics, clinical features, technical findings, treatment modalities, and outcomes were collected from the electronic patient files. A follow-up was performed via telephone interviews and clinical visits.

**Results:**

Of 149 patients, 17% had AE, 73% IE, and 10% encephalitis of unknown etiology. Significant differences between AE and IE included the prevalence of acute symptomatic seizures (AE: 85% vs. IE: 20%, *p* < 0.001), fever (8% vs. 72%, *p* < 0.001), headache (15% vs. 61%, *p* < 0.001), and focal neurological deficits (56% vs. 23%, *p* = 0.004), respectively. Paraclinical differences comprised lower CSF pleocytosis in AE compared to IE (median 6 cells/µl vs. 125 cells/µl, *p* < 0.001). Epileptic discharges on EEG and MRI lesions were more prevalent in AE than IE (50% vs. 14%, *p* < 0.001; 50% vs. 28%, *p* = 0.037). The modified Rankin Scale scores at discharge and last follow-up (median duration 2304 days, IQR 1433–3274) indicated favorable outcomes in both groups.

**Conclusion:**

This comprehensive analysis provides insights into the epidemiology, clinical, paraclinical, and therapeutic aspects and the outcomes of AE and IE in adults. We developed a diagnostic tool that facilitates early differentiation between AE and IE, aiding in timely therapeutic decision-making.

**Supplementary Information:**

The online version contains supplementary material available at 10.1007/s00415-024-12712-7.

## Introduction

Encephalitis refers to inflammation of the brain with typically autoimmunological or infectious—often viral—etiology. Both are highly dynamic entities due to the rapid discovery of novel autoantibodies over the past 2 decades and because of emerging infectious diseases [1, 2]. Encephalitis causes significant morbidity and is frequently associated with persisting neurological deficits, cognitive and neuropsychiatric symptoms and signs, and structural or immune-mediated epilepsy [3–7].

The diagnosis of autoimmune encephalitis (AE) may prove challenging due to a high rate of unremarkable magnetic resonance imaging (MRI) and cerebrospinal fluid (CSF) analyses [8, 9]. Furthermore, approximately 50% of all AE patients are considered antibody-negative [10]. On the other hand, paraclinical tests may also be negative or normal in infectious encephalitis (IE), particularly in the immunocompromised [11, 12]. In up to 60% of IE patients, the causative pathogen cannot be identified [13]. Altogether, the etiology of all types of encephalitis remains unresolved in ~ 50% [14, 15]. These diagnostic difficulties pose a dilemma as early institution of therapy is associated with a more favorable outcome [16–20], but AE and IE require different therapeutic strategies.

Prognosis varies widely for different subtypes of AE and IE [21–23]. Besides intrinsic characteristics of the specific disease, this may be due to diverse diagnostic and therapeutic approaches within different healthcare settings or even between various hospitals within the same healthcare system. Since most studies focus on either AE or IE or even on a single subcategory, it is challenging to compare the characteristics and prognosis of autoinflammatory and infectious encephalitis.

We aim to close this gap by investigating and comparing symptoms, signs, and paraclinical test results of AE and IE patients treated at three neurology departments of university hospitals within a 10-year period in a combined retro- and prospective approach. Furthermore, we develop a statistical tool to discriminate between AE and IE early in the course of the disease.

## Methods

### Study population and data extraction

All patients with an ICD-10 diagnosis consistent with encephalitis (Supplemental File 1) who were treated at one of the participating centers (Kepler University Hospital Linz (Austria)—Department of Neurology 1, Kepler University Hospital Linz (Austria)—Department of Neurology 2, Paracelsus Medical University, Salzburg (Austria)—Department of Neurology; the two departments in Linz are located at different sites and serve different catchment areas of the city of Linz and the State of Upper Austria) between January 1st 2007 and July 31th 2017 were screened. The study was approved by the ethics committees of Upper Austria (EK Nr. 1112/2018) and Salzburg (EK Nr. 415-E/2419/4-2018).

Inclusion criteria were age 18 to 99 years, at least “possible” encephalitis according to the International Encephalitis Consortium (IEC) diagnostic criteria [24], verification of well-characterized antineuronal autoantibodies or pathogens or at least “probable” autoimmune encephalitis according to Graus et al. [25]. Patients with a pre-morbid modified Rankin scale (mRS) score of 4 or more, those suffering from preexisting epilepsy, and those without follow-up were excluded.

For individual sub-analyses (discriminant analyses, bootstrapping), stricter diagnostic criteria pertained: at least “probable” autoimmune encephalitis according to the Graus criteria, at least “probable” infectious encephalitis according to the IEC criteria, at least “possible” infectious encephalitis according to the IEC criteria AND CSF pleocytosis AND detection of a relevant pathogen, or at least “possible” infectious encephalitis according to the IEC criteria AND a CSF pleocytosis of at least 50 cells/µl.

Relevant data of the initial hospitalization as well as on further hospital stays and outpatient visits were extracted from the electronic patient files (Supplemental File 1). Groups were categorized as AE or IE according to the discharge diagnosis. If a definite diagnosis could not be made at the time of discharge, an experienced neurologist reviewed the entire patient file including further diagnostic results and the clinical course (monophasic vs. remittent). Individuals in whom the etiology of encephalitis could not definitely be specified as AE or IE were diagnosed as encephalitis of unknown etiology (EUE).

Immunocompromising disease was defined as active malignant disease, ongoing immunosuppressive therapy, active human immunodeficiency virus (HIV) infection, congenital immunodeficiency, post-splenectomy, or leukopenia < 500/µl. Fever was defined as body temperature > 38 °C.

### Prospective follow-up

All eligible patients were sent a letter explaining the study in detail. They were informed that a telephone interview was planned, at the beginning of which they would be asked whether they would like to take part in the study. If the patient consented, a structured telephone interview was conducted (Supplemental File 2). In summary, the interviewer asked about persisting neurological deficits, current limitations concerning activities of daily living according to the mRS [26] and events suggestive of epileptic seizures. The latter were screened for using a validated questionnaire [27]. Diagnosis of epilepsy was made according to the International League Against Epilepsy (ILAE) criteria [28]. If the patient had deceased or was unable to conduct the interview, information was sought from a close relative or the patient’s last general practitioner. The patient’s last documented phone number was called at least three times on different days at different times before the patient was declared lost to follow-up. During the telephone interview, patients were invited for a clinical visit and instructed to take a caregiver with them to this visit if possible. This visit was conducted by an experienced neurologist and included a detailed personal and third-party medical history, a neurological examination and determination of the current mRS score (Supplemental File 3). Further examinations such as EEG, laboratory investigations or cognitive screening tests were performed as needed in case of diagnostic uncertainty.

### Statistics

All data of continuous variables were checked for normal distribution (test of normality: Kolmogorov–Smirnov with Lilliefors significance correction, type I error = 10%) and variance homogeneity (Levene’s test, type I error = 5%). Continuous variance homogenous variables with normally distributed data were compared by Student’s t-test for independent samples. Continuous variance heterogenous variables with normally distributed data were compared by Welch’ *t*-test for independent samples. For comparisons of continuous variables without normally distributed data and of variables measured on ordinal scales, the exact Mann–Whitney *U* test was used. Dichotomous variables were compared by the Fisher’s exact test, the other categorical variables by the exact Chi-square test. Correlations between dichotomous variables and variables measured on ordinal scales as well as (always not normally distributed) continuous variables were reviewed by point biserial Spearman’s rank correlation coefficients. Associations of dichotomous variables were investigated by Phi coefficients (combined with Fisher’s exact test).

A discriminant analysis was carried out to assign the cases with the encephalitis etiology “unclear” to the two etiologies “autoimmune” and “infectious”. The classification variables are listed in the results section. To assess the robustness of the results of the discriminant analysis bias-corrected and accelerated 95% confidence intervals based on 2000 bootstrapping samples were calculated for the linear discriminant functions according to Fisher. The type I error was not adjusted for multiple testing. Therefore, the results of inferential statistics are descriptive only. Statistical analyses were performed using the open-source R statistical software package, version 4.2.3 (The R Foundation for Statistical Computing, Vienna, Austria). Statistics were performed by Wolfgang Schimetta, Department of Applied Systems Research and Statistics, Johannes Kepler University, Linz, Austria. Data are reported according to RECORD guidelines for observational studies using routinely collected health data.

## Results

### Study population

**Fig. 1:**
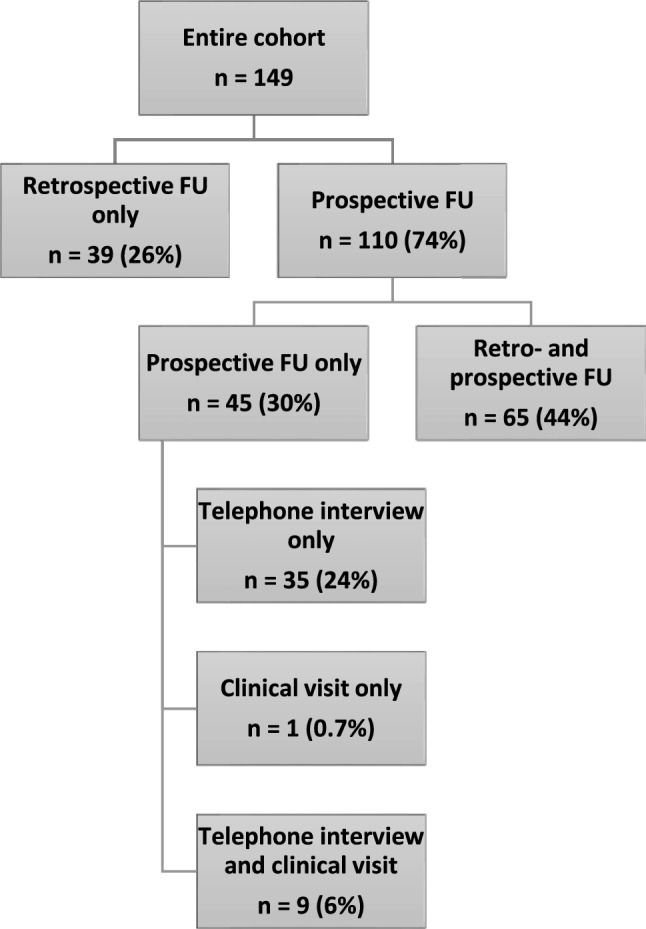
Follow-up of patients included in this cohort. *FU* Follow-up. Percentages relate to the entire cohort of *n* = 149 patients

One hundred and sixty patients with an ICD 10 diagnosis consistent with encephalitis were screened for eligibility. Of these, 149 patients (80 males, 54%) fulfilled the inclusion criteria. The majority (*n* = 110, 74%) had a prospective follow-up, while 39 (26%) individuals had their last follow-up purely retrospectively via electronic patient file analysis (Fig. 1).

**Fig. 2:**
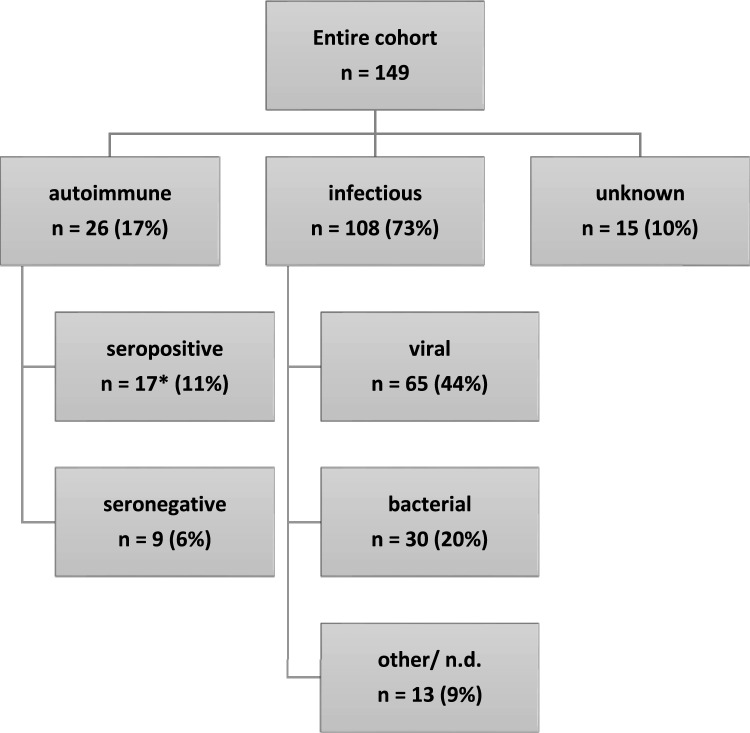
Encephalitis etiology in the investigated cohort. *CASPR2 (*n* = 5), LGI1 (4), NMDAR (2), SOX1/Amphiphysin (1), GABA_B_ (2), GAD (1), Ma2 (1), AMPA (1). Viral pathogens: tick-borne encephalitis virus (26), herpes simplex virus type 1 (9), varicella zoster virus (2), Epstein–Barr virus (2), influenza A virus (2), cytomegalovirus (1), measles virus (1), enterovirus (1), undetermined (21). Bacterial pathogens: *Streptococcus pneumoniae* (10), other *Streptococcus* species (4), *Listeria monocytogenes* (4), *Neisseria meningitidis* (2), *Staphylococcus aureus* (2), undetermined (8). Other/undetermined infectious pathogen (13)

Overall, 108 (73%) patients were diagnosed with infectious and 26 (17%) with autoimmune encephalitis. The remaining 15 individuals were categorized as EUE. Definite causative antibodies or pathogens were diagnosed in 17 (65%) and 68 (63%), respectively (Fig. 2). The median age at symptom onset was 59 years (range 21–77) for AE, 63 years (range 20–92) for IE, and 56 years (range 23–78) for EUE (15 patients). Median delay between first symptoms and hospitalization was 2 days for each AE (IQR 0–76) and IE (IQR 0–5). Median length of hospital stay was 22 days (IQR 11–52) for AE and 20 days (IQR 14–39) for IE. Median duration of follow-up from first symptoms was 1761 days for AE (IQR 1058–2244) and 2585 days for IE (IQR 1459–3335).

For the discriminant and bootstrapping analysis, six individuals were excluded who did not fulfill the stricter inclusion criteria (3 male, 6 IE).

### Clinical characteristics

In total, 15 (10%) patients were immunocompromised at the time of diagnosis: 3 (12%) in the AE, 11 (10%) in the IE, and 1 (7%) in the EUE group. Fifteen (58%) AE and 108 (100%) IE patients presented with quantitative or qualitative impairment of consciousness (*p* < 0.001). Acute symptomatic seizures occurred more frequently in AE (*n* = 22, 85%) compared to IE (*n* = 22, 20%; *p* < 0.001). Among the AE cohort, seven (27%) presented with status epilepticus, while this occurred in only three (3%) IE patients (*p* < 0.001).

Cognitive deficits including—but not limited to—impairments of attention, memory, and executive function at admission for initial hospitalization were reported in 22 (84%) AE and 96 (89%) IE patients. Fever was present in 2 (8%) AE and 78 (72%) IE patients (*p* < 0.001). Headache was more prominent among IE (*n* = 66; 61%) than among AE patients (*n* = 4; 15%, *p* < 0.001). Three (12%; AE) and eight (7%; IE) displayed autonomic symptoms. Focal neurological deficits at initial presentation were more common in IE (*n* = 60; 56%) than in AE (*n* = 6; 23%; *p* = 0.004). Among the most common symptoms ranked aphasia (*n* = 4; 15%) and motor deficits (*n* = 2; 8%) in AE, and aphasia (*n* = 32; 30%), motor deficits (*n* = 17; 16%), cerebellar/extrapyramidal symptoms (*n* = 13; 12%), and brainstem/cranial nerve lesions (*n* = 11; 10%) in IE. Neurological deficits could be verified during the clinical visit for the cranial nerves (3 patients), the motor system including coordination (9 patients), the sensory system (3 patients), as well as for language and cognition (7 patients).

**Fig. 3:**
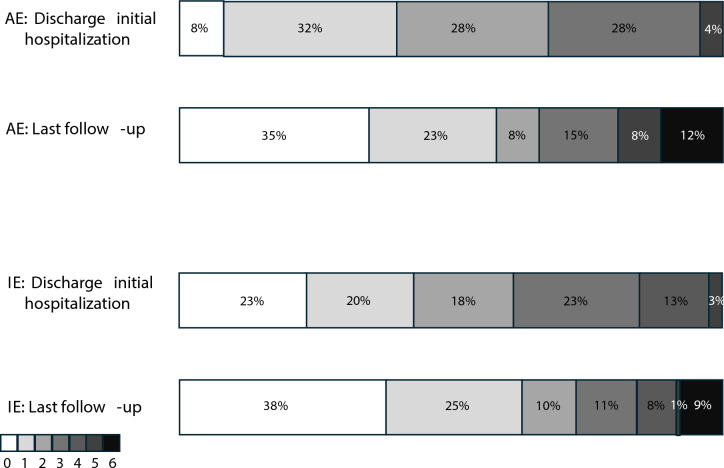
Distribution of mRS scores at hospital discharge and at last follow-up for AE and IE. *AE* autoimmune encephalitis, *IE* infectious encephalitis, *mRS* modified Rankin Score

More IE patients (*n* = 45; 42%) than AE patients (*n* = 7; 27%) needed treatment on an intensive care unit (ICU), with 23 (21%) and 5 (20%), respectively, requiring mechanical ventilation. Reasons for ICU admission other than mechanical ventilation included cardiovascular instability with the need to apply catecholamines, high risk of respiratory complications and renal replacement therapy. The distribution of mRS scores at hospital discharge and at last follow-up are presented in Fig. 3. At last follow-up available, 6 (23%) AE and 12 (11%) IE patients remained with focal neurological deficits.

### Paraclinical characteristics

Details on cerebrospinal fluid (CSF) analysis were available for 148 individuals (99%). For one patient, the diagnosis of “CSF pleocytosis”, but no cell count, total protein, etc. were available. AE and IE differed significantly concerning the degree of pleocytosis (AE: median 6 cells/µl, IQR 1–11; IE: median 125 cells/µl, IQR 52–347; *p* < 0.001). Further significant CSF and serum parameters are displayed in Table 1.

**Table 1:** CSF and serum parameters at initial hospitalization due to encephalitis (median; range)

	AE	IE	*p*-value
**CSF**			
Leukocytes (*n*/µl)	6 (0; 200)	125 (1; 40,960)	< 0.001
Granulocytes (%)	0 (0; 80)	21 (0; 100)	< 0.001
Total protein (mg/dl)	38 (18; 427)	91 (25; 3888)	< 0.001
**Serum**			
Highest CRP (mg/dl)	1.18 (0; 17.3)	5.7 (0.03; 53.11)	0.002
Lowest platelets (G/l)	210 (120; 325)	176 (28; 1234)	0.043
Lowest sodium (mmol/l)	137 (120; 145)	134 (97; 148)	0.005
TSH (µU/ml)	2.27 (0.18; 22.29)	0.87 (0.06; 16.77)	< 0.001

*AE* autoimmune encephalitis, *IE* infectious encephalitis, *CSF* cerebrospinal fluid, *CRP* C-reactive protein, *TSH* thyroid-stimulating hormone

134 patients (90%) received an EEG during their initial hospital stay. Epileptic discharges and/or ictal patterns were more commonly found in AE (*n* = 13, 50%) compared to IE (*n* = 14, 14%; *p* < 0.001).

Lesions on fluid-attenuated inversion recovery (FLAIR)-/T2-weighted cerebral MRI were present in 13 (50%) patients with AE and 30 (28%) patients with IE (*p* = 0.037). In AE, by far the most common affected site was the mesio-temporal lobe (92%), while in IE, lesions were detected in the mesio-temporal lobe (55%), the extratemporal supratentorial region (41%), and the basal ganglia (31%). Contrast-enhancing lesions were reported in 13% of AE and 12% of IE.

### Therapy

During initial hospitalization, 7 (27%) AE and 95 (88%) IE patients received antibiotics, while 8 (31%) and 77 (71%) were treated with virostatic medication, most frequently acyclovir. Immunosuppressive treatment was initiated in 21 (81%) AE patients (steroids *n* = 15, intravenous immunoglobulins (IVIG) *n* = 14, plasmapheresis *n* = 5, rituximab (RTX) *n* = 1) and 15 (14%) IE patients (steroids *n* = 15, IVIG *n* = 3). At last follow-up, five (19%) individuals with AE continued receiving immunosuppressive treatment. The median delay from symptom onset to the initiation of immunosuppressive therapy in AE was 36 days (IQR 6–92), with a median delay of 2 days from hospitalization to treatment commencement (IQR 0–76).

### Discriminant analysis for encephalitis of unknown etiology

In an attempt to classify the individuals considered as EUE as either AE or IE, a discriminant analysis was conducted using variables that had displayed significant differences between those two entities to develop a well-fitting model:

- Presence of disorders of consciousness at initial presentation (favors IE).
- Presence of acute symptomatic seizures at initial presentation (favors AE).
- Presence of headache at initial presentation (favors IE).
- Presence of fever (> 38 °C) at initial presentation (favors IE).
- CSF: presence of pleocytosis (favors IE).
- CSF: leukocyte count (higher count favors IE).

Of the 15 EUE cases, 12 could be grouped as IE with a likelihood of 90.04 to 99.83%. Two of the remaining individuals were most likely to have AE (70.13%, 70.61%). One case could not be assigned. The fit of the model was demonstrated by a Wilks’ lambda of 0.399 (*p* < 0.001). The results of bootstrapping are listed in Supplemental File 5. The statistical tool used is included in this publication as an interactive Excel spreadsheet for clinicians’ use (Supplemental File 4).

## Discussion

This paper proposes a detailed characterization of AE and IE patients. We also developed a diagnostic tool to improve early diagnosis of encephalitis and hence accelerate adequate therapy in these patients.

### Epidemiology and (para-)clinical patient characteristics

In our cohort, women and men were evenly represented. IE occurred approximately four times more frequently than AE, a reflection of the general incidence rates of these diseases [29–31]. Strict criteria were applied to ensure the inclusion of only those cases with a high level of confidence in the diagnosis of encephalitis and its classification as either IE or AE. In particular, unspecific syndromes accompanied by antibodies with low diagnostic value were excluded, as these cases are prone to overdiagnosis of AE [32, 33]. The distribution of AE subgroups is consistent with findings from other studies, with CASPR2, LGI1, and antibody-negative AE being the most frequently encountered entities in adults [30, 34, 35]. The spectrum of pathogens in IE reflects the regionality with a high prevalence of tick-borne encephalitis virus (TBEV), an entity with increasing prevalence due to global warming [36].

Most patients were hospitalized promptly after the onset of symptoms. However, some individuals—particularly in AE—presented only after a significant delay. In most of these cases, encephalitis was preceded by symptoms not easily recognized as signs of CNS inflammation, such as subtle faciobrachial dystonic seizures and depression, which may have been misinterpreted by both the patients and primary care physicians. The most common presenting symptoms were disorders of consciousness and cognitive deficits. This is unsurprising, as alterations of consciousness are included in the diagnostic criteria of the International Encephalitis Consortium, and cognitive impairments are considered a hallmark of AE [24, 37]. Additional symptoms, particularly acute symptomatic seizures, fever, and headache, have been found to effectively differentiate between AE and IE, with the former favoring AE and the latter two IE [29, 38]. Our study reaffirms their utility in distinguishing between the two conditions: a well-fitted model incorporating these as well as CSF pleocytosis/leukocyte count allowed for confident assignment of 93% of EUE cases to either IE or AE.

Focal neurological deficits were more prevalent in IE compared to AE. While AE commonly presents with the typical triad of limbic encephalitis, consisting of cognitive deficits, epileptic seizures, and psychiatric disorders, focal deficits are less frequent [37]. This may be due to the mesiotemporal pathology seen in many AE. The requirement for ICU treatment and mechanical ventilation was high in both patient subgroups. Some studies have linked these features to a poorer outcome [18, 39]. However, we did not observe a correlation with the overall outcome as measured by the mRS score. This unexpected result may have been influenced by a selection bias as multimorbid patients with severe encephalitis might not have been referred to intensive care in the first place.

Significant differences in laboratory parameters between AE and IE include CSF leukocyte count, as well as serum C-reactive protein (CRP), platelets, and sodium. In AE, reports have indicated either normal or only mildly elevated CSF leukocytes [12, 29, 31], with varying proposed cutoffs between 36 and 50 cells/µl to differentiate AE from IE [38, 40]. Lower serum sodium and platelets levels in IE compared to AE have been previously noted, possibly reflecting a more pronounced systemic reaction to infectious pathogens [29, 38]. These observations extend to clinical features such as headache and fever, which we found to be discriminative between the two conditions.

Characteristic MRI patterns are often absent in many cases of both AE and IE [31, 41]. However, among MRI-positive cases, there was a high prevalence of cortical and mesio-temporal lesions in AE. This can be attributed to the pathophysiology of AE, with limbic encephalitis ranking among the most common syndromes [37, 42]. Due to the high prevalence of TBE in our sample, basal ganglia and infratentorial lesions prevailed in IE [43].

Rates of encephalitis due to undetermined etiology remain high in various studies [29, 31]. Beyond the risk of introducing classification bias into research focused on this entity, this diagnostic uncertainty also proves challenging for clinical practice. Regarding our cohort, we were able to determine six discriminating factors that can easily be obtained early in the course of the disease: disorders of consciousness (favors IE), acute symptomatic seizures (favors AE), headache (favors IE), fever (favors IE), and CSF pleocytosis including leukocyte count (favors IE). On this basis, all but one EUE case could be assigned to either AE or IE. Bootstrapping was performed for internal validation of the discriminant analysis. Since the bootstrap confidence intervals are relatively wide, it is important to head the probabilities assigned to either IE or AE in the diagnostic tool and apply a clinical plausibility check, particularly when the probabilities approach 50% or the differential diagnosis concerns a highly unusual case.

### Therapy and outcome

The therapeutic regimes administered to our patients adhere to the clinical practice guidelines on AE/IE [32, 44, 45]. The limited use of RTX may reflect the time of patient recruitment, as RTX has only recently been proposed as a treatment option in the initial therapy of severe AE. The add-on therapy with steroids and IVIG in IE may have been prompted by initial uncertainty regarding the pathogenesis of the encephalitis or could represent an anti-inflammatory approach to IE [46, 47].

Overall, the general outcome for patients with IE and AE was favorable, with 71% of all patients achieving an mRS of 0–2 at last follow-up (AE 66%, IE 73%). Within this group of patients with good outcomes, there was further improvement between initial discharge and last follow-up as deduced from a shift towards mRS scores of 0 and 1. These findings are consistent with previous reports indicating a favorable outcome in 80 to 100% of AE patients [39, 48] and approximately 50% in IE [49]. However, as we only included patients with a follow-up, initial mortality and morbidity is underestimated in our sample. Furthermore, the mRS does not capture the impact of neuropsychological and cognitive deficits on quality of life [42, 48, 50]. Hence, more specific encephalitis scores such as the CASE score or patient-reported outcome measures should be incorporated into studies on encephalitis outcome [51, 52].

Advantages of our study include the large number of patients, the multicentric approach, and the opportunity for comparison of IE and AE characteristics and outcome within the same healthcare setting. Shortcomings of our study include the heterogeneity of our patient cohort concerning the causative pathology. This may have been further exacerbated by the extended recruitment and prolonged follow-up period which was partially due to the Covid-19 pandemic. While most patients had a prospective follow-up, only retrospective data were available in one quarter of the cohort. However, we still chose to include these patients to avoid bias towards patients with less severe clinical courses as among the main reasons participants could not be contacted prospectively were premature death or severe disability.

In summary, we present a comprehensive retro- and prospective analysis of the characteristics, outcome and differentiating factors of a sizable cohort comprising patients with AE and IE. Key findings from our investigation include:

- Most frequent encephalitis etiology in an adult European cohort comprises seronegative and CASPR2-antibody-positive AE, and TBEV and S. pneumonia (IE).
- Despite the often severe symptoms requiring ICU management, the majority of encephalitis patients experience resolution of focal neurological deficits and significant improvement in their activities of daily living (mRS) at hospital discharge compared to admission. Further improvement can be expected after hospital discharge.
- We integrate six readily accessible clinical and CSF parameters (impairment of consciousness, acute symptomatic seizures, headache, fever, and CSF pleocytosis including leukocyte count) into a discriminative model for timely differentiation between AE and IE and provide a calculation tool for respective diagnostic probabilities. This tool will support clinicians in early decision making as to the therapeutic approach.

## Supplementary information

Below is the link to the electronic supplementary material.

Supplementary file1 (DOCX 16 KB)

Supplementary file2 (DOCX 24 KB)

Supplementary file3 (DOCX 25 KB)

Supplementary file4 (XLSX 15 KB)

Supplementary file5 (DOCX 34 KB)
