# Supplementary material for: Autoimmune and infectious encephalitis: development of a discriminative tool for early diagnosis and initiation of therapy
Source: J Neurol. 2024 Oct 5;271(12):7583–91. doi: 10.1007/s00415-024-12712-7 (PMC11588785; doi:10.1007/s00415-024-12712-7)
Supplement: Supplementary file 1 — Supplementary file1 (DOCX 16 KB) [file 415_2024_12712_MOESM1_ESM.docx]

**Autoimmune and infectious encephalitis: development of a discriminative tool for early diagnosis and initiation of therapy**

Journal of Neurology

Tobias Moser, Joachim Gruber, Eirini Mylonaki, Vincent Böhm, Daniel Schwarzenhofer, Anna R. Tröscher, Eva Lenzenweger, Ingomar Krehan, Eva Söllradl, Markus Leitinger, Raimund Helbok, Eugen Trinka, Tim J. von Oertzen, and Judith N. Wagner

**Corresponding author:**

Judith N Wagner, MD

Department of Neurology, Evangelisches Klinikum Gelsenkirchen

Teaching Hospital University Duisburg-Essen

Munckelstrasse 27, 45879 Gelsenkirchen, Germany

Tel. +49 (0)209 160 -1501, Fax +49 (0)209 160 -2684

judith.wagner@evk-ge.de

ORCID 0000-0002-0776-6821

**RELEVANT ICD 10 CODES**

G04.0, G04.8, G04.9, G05.1, G05.2, G05.8, A81.1/2/8/9, A83.0-9, A84.0/1/8/9, A85.0, A85.1, A85.2, A85.8, A86, A89, A97.2/9, B00.4, B05.0, B26.2, B06.0, B01.1, B02.0, B25.88, J10.8, J09, B05.1, A02.0, A17.0, A17.8, A32.1, A87.-, G00.-, G01.-, G02.-, G03.-, G37.8

**DATA COLLECTD_SCREENING**

- Age
- Sex
- Alteration of consciousness
- Fever > 38°C
- Acute symptomatic seizures
- New neurological deficits
- CSF leukocytosis
- New MRI or CT lesions compatible with encephalitis
- EEG alterations compatible with encephalitis
- Exclusion of relevant differential diagnoses

**DATA COLLECTD_INITIAL HOSPITALIZATION**

- Date of birth
- Sex
- Date first symptoms
- Date first hospitalization
- Date extraction patient data
- Etiology encephalitis
- Definite diagnosis (specific pathogen or antibody)
- Certainty of diagnosis
- Interval first symptoms – initiation of therapy (for AE)
- Level of consciousness
- New neurological deficit (yes vs. no; if yes – which)
- Other symptoms (headache, autonomous symptoms, neuropsychological symptoms)
- MRI/CT lesion compatible with encephalitis (constrast-enhancement? Gray vs. white matter? Topographic localization? Cerebral edema?)
- Structural lesion independent of encephalitis (which? Potentially epileptogenic?)
- Acute symptomatic seizures (semiology?)
- Status epilepticus (semiology?)
- EEG: epileptiform discharges? Seizure patterns?
- Antiseizure medication (which? Maximal dose? Effect on seizure activity?)
- Antibacterial/ antiviral/ immunosuppressive therapy
- Comorbidity, particularly immunosuppressive disease
- Need for ICU-treatment or mechanical ventilation
- Date first CSF analysis
- Results first CSF analysis (pleocytosis, leukocyte count, leukocyte differentiation, oligoclonal bands, intrathecal immunoglobulin synthesis, CSF/serum glucose ratio, protein)
- Highest serum CRP
- Lowest thrombocyte count
- Lowest serum sodium
- TSH
- mRS score at discharge

**DATA COLLECTD_RETROSPECTIVE FOLLOW-UP**

- Date of birth
- Date last follow-up
- Date of death if patient deceased
- Date extraction patient data
- Neurological deficit (yes vs. no; if yes – which)
- Status epilepticus (semiology?)
- Antiseizure medication (which? Maximal dose? Effect on seizure activity? Side effects? Duration of therapy/ ongoing?)
- Immunosuppressive thrapy (which? Maximal dose? Duration of therapy/ ongoing?)
- Postencephalitic epilepsy (PEE; first PEE-defining seizure, semiology, frequency)
- New structural lesion independent of encephalitis
- Encephalitis relapse or ongoing disease
- mRS score

**DATA COLLECTD_PROSPECTIVE FOLLOW-UP: TELEPHONE INTERVIEW**

- Date follow-up
- Date of death if patient deceased
- Neurological or cognitive deficit (yes vs. no; if yes – which)
- Postencephalitic epilepsy (PEE; first PEE-defining seizure, semiology, frequency)
- Status epilepticus (semiology?)
- Antiseizure medication (which? Dose? Effect on seizure activity? Side effects? Duration of therapy/ ongoing?)
- Immunosuppressive therapy
- New structural lesion independent of encephalitis
- New relevant morbidity
- mRS score

**DATA COLLECTD_PROSPECTIVE FOLLOW-UP: CLINICAL VISIT**

- Date follow-up
- Neurological or cognitive deficit (yes vs. no; if yes – which; results of physical neurological examination)
- Postencephalitic epilepsy (PEE; first PEE-defining seizure, semiology, frequency)
- Status epilepticus (semiology?)
- Antiseizure medication (which? Dose? Effect on seizure activity? Side effects? Duration of therapy/ ongoing?)
- Immunosuppressive therapy
- New structural lesion independent of encephalitis
- New relevant morbidity
- mRS score
- presence of caregiver
