# Supplementary material for: Autoimmune and infectious encephalitis: development of a discriminative tool for early diagnosis and initiation of therapy
Source: J Neurol. 2024 Oct 5;271(12):7583–91. doi: 10.1007/s00415-024-12712-7 (PMC11588785; doi:10.1007/s00415-024-12712-7)
Supplement: Supplementary file 3 — Supplementary file3 (DOCX 25 KB) [file 415_2024_12712_MOESM3_ESM.docx]

**Autoimmune and infectious encephalitis: development of a discriminative tool for early diagnosis and initiation of therapy**

Journal of Neurology

Tobias Moser, Joachim Gruber, Eirini Mylonaki, Vincent Böhm, Daniel Schwarzenhofer, Anna R. Tröscher, Eva Lenzenweger, Ingomar Krehan, Eva Söllradl, Markus Leitinger, Raimund Helbok, Eugen Trinka, Tim J. von Oertzen, and Judith N. Wagner

**Corresponding author:**

Judith N Wagner, MD

Department of Neurology, Evangelisches Klinikum Gelsenkirchen

Teaching Hospital University Duisburg-Essen

Munckelstrasse 27, 45879 Gelsenkirchen, Germany

Tel. +49 (0)209 160 -1501, Fax +49 (0)209 160 -2684

judith.wagner@evk-ge.de

ORCID 0000-0002-0776-6821

**Clinical visit**

**Screening number Patient:** **__________ Patient ID_______________**

**Date of birth:** **_________ m/f**

**Name of interviewer:______________________ Department/Clinic: ________________**

**Date of interview:** **____________**

**Is the patient accompanied by another person (third-person anamnesis)?** **Yes/No**

**If yes, name and relationship to the patient:** **______________________**

**Step 1**: Has the patient been able to clarify all his/her questions regarding the study and has he/she signed the consent form? **Yes/No**

(if no: clarify what information the patient needs that would allow him/her to participate in the study; if he/she definitely refuses - clarify and document whether the retrospectively collected data may be used)

**Step 2**: Go through the following questionnaires with the patient and their carer (Tables 1, 2 and 3). In the event of divergent answers regarding the telephone interview, **the results of the personal examination are decisive for the final assessment regarding the presence and characteristics of postencephalitic epilepsy!**

**Screening for epileptic seizures (Placencia et al., Brain 1992)**

|  | **Answer (please mark clearly)** | **Personal assessment: the reported event corresponds with ... certainty to an epileptic seizure (please mark clearly)** | **If the event corresponds with HIGH certainty to an epileptic seizure, adde the seizure classification according to the ILAE Seizure Classification 2017 + frequency and period of events + response to therapy + provocation factors** |
| --- | --- | --- | --- |
| Have you ever lost consciousness? | Yes / No | high/low/unclear |  |
| Have you ever had attacks in which you lose contact with the surroundings? | Yes / No | high/low/unclear |  |
| Have you ever had attacks of shaking of the arms or legs which you could not control? | Yes / No | high/low/unclear |  |
| Have you ever had attacks in which you fall to the ground with loss of consciousness? | Yes / No | high/low/unclear |  |
| Have you ever had attacks in which you fall and bite your tongue? | Yes / No | high/low/unclear |  |
| Have you ever had attacks in which you fall and lose control of your bladder? | Yes / No | high/low/unclear |  |
| Have you ever had attacks in which you fall and become pale? | Yes / No | high/low/unclear |  |
| Have you ever had attacks in which you lose your memory for a short period of time? | Yes / No | high/low/unclear |  |
| Have you ever had attacks of strange behaviour and loss of memory? | Yes / No | high/low/unclear |  |
| Have you ever had brief attacks of shaking or trembling in one arm or leg or in the face? | Yes / No | high/low/unclear |  |
| Have you ever had attacks of tingling or numbness which move up your arm, leg or body? | Yes / No | high/low/unclear |  |
| Have you ever had attacks of jerkings which move up your arm, leg or body? | Yes / No | high/low/unclear |  |
| Have you ever had attacks in which you lose contact with the surrounding and experience a feeling of unreality or dreaminess? | Yes / No | high/low/unclear |  |
| Have you ever had attacks in which you lose contact with the surrounding and experience a sensation in which objects change shape or size? | Yes / No | high/low/unclear |  |
| Have you ever had attacks in which you lose contact with the surrounding and experience abnormal visions? | Yes / No | high/low/unclear |  |
| Have you ever had attacks in which you lose contact with the surrounding and experience abnormal sounds? | Yes / No | high/low/unclear |  |
| Have you ever had attacks in which you lose contact with the surroundings and experience abnormal smells? | Yes / No | high/low/unclear |  |
| Have you ever had attacks in which you behave momentarily in a confused fashion? | Yes / No | high/low/unclear |  |
| Have you ever had attacks of palpitation? | Yes / No | high/low/unclear |  |
| Have you ever been told that you have or had epilepsy or epileptic seizures? | Yes / No | high/low/unclear |  |

**Further questions – Table 2**

|  | **Antwort (bitte eindeutig markieren)** | **Detailinformationen** |
| --- | --- | --- |
| If you answered "yes" to one of the questions in Table 1 (Screening according to Placencia et al., Brain 1992) - has a diagnosis already been made in this regard? | Yes / No | Which diagnosis? |
| If you answered "yes" to one of the questions in Table 1 (Screening according to Placencia et al., Brain 1992), have further diagnostic tests (e.g. EEG, cMRI, LP) already been carried out? | Yes / No | Which tests? Results? |
| In the case of existing epileptic seizures, collect further detailed information for each type of seizure |  | Semiology:  First-time occurrence:  Frequency (n/week): |
| Are you currently taking medication for epileptic seizures (collect detailed information for each drug)? | Yes / No | Name of drug:  Max. dose:  Start of medication:  Efficacy:  Side effects:  Reasons for potential dose reductions: |
| Have you taken any other medication for epileptic seizures in the interval since the last follow-up at our clinic (collection of detailed information for each drug)? | Yes / No | Name of drug:  Max. dose:  Start of medication:  Efficacy:  Side effects:  Reasons for discontinuation: |
| Have you ever had status epilepticus?  (If necessary, explain to patient: particularly long seizure, not self-limiting...) | Yes / No | Semiology:  When:  Duration:  Therapy (where):  Therapy (how): |
| According to the patient, is there a residual neurological deficit (outside of epilepsy)? | Yes / No | Which deficit: |
| According to the patient, has a new cerebral structural lesion occurred in the interval since the last follow-up? | Yes / No | Which lesion: |
| Does the patient have a new concomitant disease that has occurred in the interval since the last follow-up? | Yes / No | Which disease: |
| Is the patient taking immunosuppressive therapy for the indication encephalitis? | Yes / No | Name of drug:  Dose:  Start of medication: |

**Modified Rankin Scale**

0  The patient has no residual symptoms.

1  The patient has no significant disability; able to carry out all pre-stroke activities.

2  The patient has slight disability; unable to carry out all pre-stroke activities but able to look after self without daily help.

3  The patient has moderate disability; requiring some external help but able to walk without the assistance of another individual.

4  The patient has moderately severe disability; unable to walk or attend to bodily functions without assistance of another individual.

5  The patient has severe disability; bedridden, incontinent, requires continuous care.

6  The patient has expired

**Physical examination**

|  | **Answer (please mark clearly)** | **If the answer is "yes" - please specify** |
| --- | --- | --- |
| Is the patient disorientated in terms of person, place or time? | Yes / No |  |
| Does the patient suffer from a disorder of consciousness? | Yes / No |  |
| Is there an abnormal mood? | Yes / No |  |
| Is there a speech disorder? | Yes / No |  |
| Are there indications of a disorder of other higher brain functions? | Yes / No |  |
| Is there an oculomotor disorder? | Yes / No |  |
| Is there a disorder of the other cranial nerves? | Yes / No |  |
| Is there a paresis? | Yes / No |  |
| Is there a sensory disorder? | Yes / No |  |
| Is there an abnormality in the reflex status/are there pathological reflexes? | Yes / No |  |
| Is there a coordination disorder/ataxia/dysmetria? | Yes / No |  |
| Is there a history of vegetative dysfunction? | Yes / No |  |
| Is there a stance and gait disorder (specify aids if necessary)? | Yes / No |  |
| **Final assessment of the neurological syndrome syndrome** | |  |

**Final assessment of the interviewer:**

- The patient most likely suffers from postencephalitic epilepsy - yes/no (please mark clearly)
- The first seizure defining postencephalitic epilepsy occurred on ________ (date)
- Please also note the information from the retrospective data collection ant the telephone interview!
- Further investigations were carried out or scheduled for definitive categorisation (yes/no; if yes: list investigations (e.g. EEG, cMRI, …)
