# Supplementary material for: Autoimmune and infectious encephalitis: development of a discriminative tool for early diagnosis and initiation of therapy
Source: J Neurol. 2024 Oct 5;271(12):7583–91. doi: 10.1007/s00415-024-12712-7 (PMC11588785; doi:10.1007/s00415-024-12712-7)
Supplement: Supplementary file 5 — Supplementary file5 (DOCX 34 KB) [file 415_2024_12712_MOESM5_ESM.docx]

| **Bootstrapping Analysis**  **Coefficients of the canonical discriminant function** | | | | | | |
| --- | --- | --- | --- | --- | --- | --- |
| **Parameter** | **non-standardised coefficients** | **95% confidence interval** | **95% confidence interval** | **standardised coefficients** | **95% confidence interval** | |
|  |  | **lower limit** | **upper limit** |  | **lower limit** | **upper limit** |
| Initial presentation: First CSF diagnosis - leucocyte count (n/µl) | 0,000 | 0,000 | 0,000 | 0,219 | 0,029 | 0,392 |
| Initial presentation: Disorders of consciousness > 24 hours | 2,213 | 1,211 | 3,690 | 0,499 | 0,283 | 0,699 |
| Initial presentation: Fever > 38°C | 1,301 | 0,613 | 2,065 | 0,529 | 0,273 | 0,740 |
| Initial presentation: New epileptic seizures | -0,908 | -1,494 | -0,371 | -0,391 | -0,592 | -0,161 |
| Initial presentation: First CSF diagnosis - pleocytosis | 1,049 | -0,136 | 2,667 | 0,302 | -0,031 | 0,606 |
| Initial presentation: Headache | 0,268 | -0,288 | 0,767 | 0,125 | -0,140 | 0,339 |
| Constant | -3,573 | -5,000 | -2,643 | - | - | - |

| **Coefficients of the canonical discriminant classification function: Fisher linear discriminant functions per group** | | | |
| --- | --- | --- | --- |
| **AUTOIMMUNE** |  | **95% confidence interval** | |
| **Parameter** | **coefficients** | **lower limit** | **upper limit** |
| Initial presentation: First CSF diagnosis - leucocyte count (n/µl) | 0,000 | 0,000 | 0,000 |
| Initial presentation: Disorders of consciousness > 24 hours | 10,587 | 5,238 | 31,577 |
| Initial presentation: Fever > 38°C | -0,841 | -2,140 | 0,180 |
| Initial presentation: New epileptic seizures | 5,959 | 3,435 | 10,646 |
| Initial presentation: First CSF diagnosis - pleocytosis | 6,132 | 1,846 | 15,735 |
| Initial presentation: Headache | 0,849 | -0,611 | 2,456 |
| Constant | -7,833 | -12,564 | -6,081 |

| **INFECTIOUS** |  | **95% confidence interval** | |
| --- | --- | --- | --- |
| **Parameter** | **coefficients** | **lower limit** | **upper limit** |
| Initial presentation: First CSF diagnosis - leucocyte count (n/µl) | 0,000 | 0,000 | 0,001 |
| Initial presentation: Disorders of consciousness > 24 hours | 17,258 | 10,689 | 49,113 |
| Initial presentation: Fever > 38°C | 3,081 | 0,554 | 6,914 |
| Initial presentation: New epileptic seizures | 3,221 | 0,275 | 6,891 |
| Initial presentation: First CSF diagnosis - pleocytosis | 9,294 | 3,317 | 27,476 |
| Initial presentation: Headache | 1,656 | -0,665 | 4,081 |
| Constant | -15,920 | -22,446 | -14,024 |
